# Cardiomyopathy in glycogen storage diseases: diagnosis, prognosis, and advanced management

**DOI:** 10.1007/s10741-026-10648-w

**Published:** 2026-07-10

**Authors:** Umang Patel, Eren Gunes, Apaara Chawla, Kiara Ashman, Adam Taub, Tigran Arakelyan, Ariyan Madani, Krishay Patel, Quan Bui, Eric Adler

**Affiliations:** Division of Cardiovascular Medicine, University of California, San Diego, CA USA

**Keywords:** Glycogen storage disease, Cardiomyopathy, Danon disease, Pompe disease, PRKAG2 cardiomyopathy, Gene therapy

## Abstract

Glycogen storage diseases (GSDs) are a heterogeneous group of rare and often under-recognized causes of inherited cardiomyopathy characterized by pathological glycogen or autophagic vacuole accumulation of debris within various tissues, including the heart. These diseases often present as phenocopies of sarcomeric hypertrophic cardiomyopathy, though they may also manifest with dilated or mixed phenotypes. Key GSDs with cardiomyopathy include Pompe disease (GSD IIa), Danon disease (IIb), Cori/Forbes disease (GSD III), Andersen disease (GSD IV), Tarui disease (GSD VII), phosphorylase kinase deficiency (GSD IX), glycogenin-1 deficiency (GSD XV), and PRKAG2 syndrome, each presenting unique clinical trajectories. Accurate diagnosis requires integration of clinical red flags, multimodality cardiac imaging, and electrocardiography, alongside definitive diagnostic tools like enzyme screening, genetic testing, and endomyocardial biopsy. There is a dearth of evidence regarding specific treatment of each unique GSD cardiomyopathy, but emerging therapeutics across the spectrum of GSDs aim to address this need. This review covers current knowledge on the spectrum of GSD-related cardiomyopathies, discussing pathophysiology, diagnosis, and evolving treatment strategies.

## Introduction

 Glycogen storage diseases (GSD) comprise a molecularly heterogeneous but clinically overlapping group of conditions that frequently result in hypertrophic cardiomyopathy (HCM) phenocopies [1, 2]. Unlike sarcomeric HCM, caused by mutations in contractile protein genes, metabolic cardiomyopathies driven by glycogen accumulation are distinct in their natural histories, extracardiac disease phenotypes, and treatment strategies [3, 4].

GSDs are caused by defects in enzymes or regulatory proteins governing glycogen synthesis, degradation, lysosomal processing, or autophagy. Those with cardiac involvement include Pompe disease (GSD IIa), Danon disease (IIb), Cori/Forbes disease (GSD III), Andersen disease (GSD IV), Tarui disease (GSD VII), phosphorylase kinase deficiency (GSD IX), glycogenin-1 deficiency (GSD XV), and PRKAG2 syndrome [1]. Each condition presents with a specific cardiac profile and multisystem involvement, but only a subset has available disease-specific therapy.

Despite advances in genomic screening and multimodal cardiac imaging, metabolic cardiomyopathies remain frequently underrecognized [4, 5]. This review provides a comprehensive evaluation of the molecular pathogenesis of GSDs with cardiac manifestations, emphasizing recent advances in diagnosis, treatment, and emerging precision therapies.

## Diagnostic approach to glycogen storage diseases

**Fig. 1:**
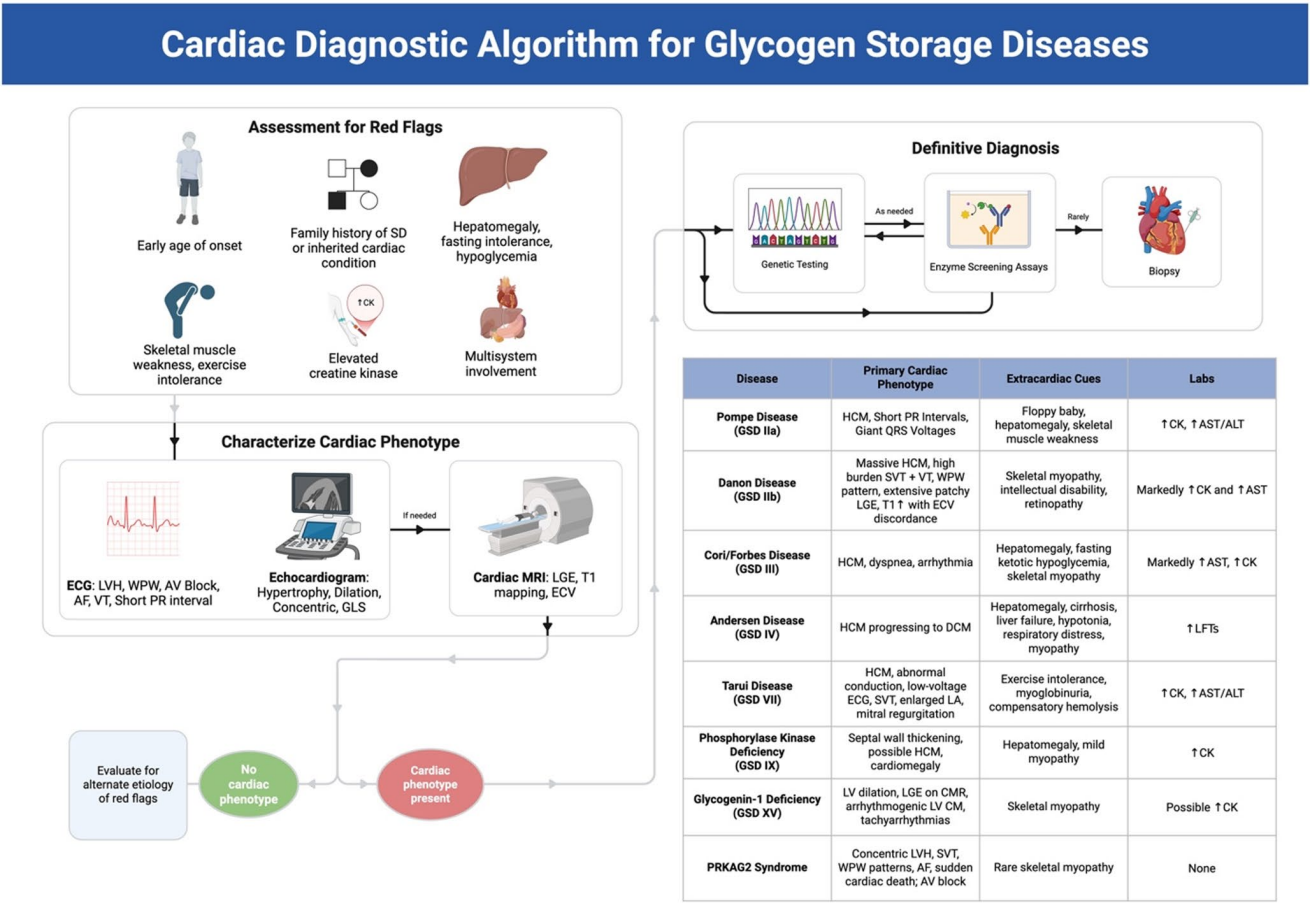
Diagnostic algorithm for glycogen storage diseases in patients presenting with cardiomyopathy. This figure presents a structured approach to identifying GSDs in patients. After a primary care physician identifies key red flags, the baseline cardiac phenotype is characterized using an electrocardiogram and echocardiogram. If needed, advanced imaging like cardiac magnetic resonance imaging is used. The presence of a cardiac phenotype alongside red flags raises suspicion for cardiomyopathy of metabolic origin, thus prompting use of definitive diagnostic tests like molecular genetic testing and enzyme screening assays. Consensus on which to use first has not been established, but at our center, use of molecular genetic testing as a first-line screening, once GSD is suspected, is preferred. Endomyocardial biopsy is used in rare cases to confirm a diagnosis. Created in BioRender. B, A. (2026) https://BioRender.com/n85hjyv. Abbreviation: ECG = electrocardiogram, WPW = Wolff Parkinson White Pattern; AV Block = atrioventricular block; AF = atrial fibrillation; VT = ventricular tachycardia; GLS = global longitudinal strain; MRI = magnetic resonance imaging; LGE = late gadolinium enhancement; ECV = extracellular volume; SD = sudden death; HCM = hypertrophic cardiomyopathy; CK = creatine kinase; AST = aspartate aminotransferase; ALT = alanine aminotransferase; SVT = supraventricular tachycardia; DCM = dilated cardiomyopathy; LA = left atrium; LV = left ventricle; CMR = cardiac magnetic resonance imaging; LV CM = left ventricular cardiomyopathy; LVH = left ventricular hypertrophy

The diagnosis of GSD cardiomyopathy requires a systematic, multimodal approach integrating clinical assessment, cardiac imaging, electrocardiography, biochemical screening, genetic testing, and, in selected cases, tissue biopsy. To facilitate clinical application, the diagnostic workup is organized into three hierarchical tiers: (2.1) Initial Evaluation, which primarily consists of identification of key GSD red flags by a primary care physician; (2.2) Cardiac Phenotype Characterization, encompassing electrocardiographic assessment and baseline imaging, biochemical investigations; and (2.3) Definitive Testing, including enzyme screening assays, genetic testing, and tissue biopsy. This tiered approach enables clinicians to systematically narrow the differential diagnosis while reserving invasive and costly investigations for appropriately selected patients. A detailed diagnostic approach is discussed in Fig. 1.

### Initial evaluation of clinical red flags

The baseline physical assessment is a critical step in the diagnosis of GSDs. Identification of clinical red flags that can distinguish GSD cardiomyopathy from sarcomeric cardiomyopathy (CM) (Fig. 1). The most important clues for the indication of a GSD are early age of symptom onset (particularly childhood or adolescence), rapidly progressive disease trajectory, skeletal muscle weakness, or exercise intolerance [2]. GSD severity varies by subtype, but it is commonly associated with hypoglycemia, hyperuricemia, hyperlactatemia, and dyslipidemia [6]. Hepatomegaly and liver fibrosis or cirrhosis are also common in certain subtypes. Muscle GSDs may present with exercise intolerance, muscle cramps/pain, rhabdomyolysis, and muscle weakness [1]. However, because initial presenting symptoms can occur in childhood or be delayed until adulthood, GSD cardiomyopathies should remain in the differential diagnosis for both pediatric and adult patients presenting with unexplained cardiomyopathy [4, 6].

A critical principle distinguishing GSD-related cardiomyopathy from sarcomeric CM is the consistent presence of multisystem organ involvement [1, 4, 7]. The pleiotropic nature of these conditions is often initially overlooked when a patient is misdiagnosed with sarcomeric CM [2].

### Cardiac phenotype characterization

#### Electrocardiographic assessment, arrhythmias and conduction abnormalities

Electrical abnormalities are a defining feature of glycogen storage cardiomyopathies. The electrocardiogram (ECG) is therefore a critical diagnostic tool to help distinguish between sarcomeric CM and GSD cardiomyopathy [2]. In sarcomeric CM, ECG abnormalities primarily reflect structural myocardial remodeling and increased left ventricle (LV) mass [6, 8]. By contrast, GSD cardiomyopathies are uniquely characterized by an array of electrophysiological abnormalities that reflect glycogen-mediated disruptions of the cardiac conduction system [1, 4].

An important limitation is that ventricular preexcitation and electrical abnormalities are not universal features of GSD cardiomyopathy. In fact, GSD III, GSD IV, and GSD XV do not typically produce preexcitation, occasionally presenting with left ventricular hypertrophy (LVH)-only or a normal ECG. These features can often delay the diagnosis of a GSD [9–11].

The ECG should therefore be interpreted as a disease-filtering rather than disease-confirming tool that can be used within a diagnostic algorithm, not in isolation.

#### Echocardiography

Echocardiography remains the first-line imaging modality. Key parameters include (LV) wall thickness, distinction between HCM and dilated cardiomyopathy (DCM) phenotype, degree and distribution of hypertrophy (concentric vs. asymmetric), LV outflow tract gradient, ejection fraction (EF), and diastolic function indices [12].

Analysis of the distribution of hypertrophy is a useful metric to diagnose certain GSDs. GSD-derived cardiomyopathies, particularly PRKAG2 syndrome and Danon disease, tend to produce concentric LVH more frequently than the asymmetric septal hypertrophy that is the most common morphological pattern in sarcomeric HCM [5, 6, 13]. This distinction is probabilistic rather than absolute. Sarcomeric HCM itself exhibits considerable morphological heterogeneity; concentric, apical, and diffuse patterns are well-recognized phenotypes, and concentric LVH can also be present in sarcomeric HCM [6, 14]. Conversely, not all GSD subtypes produce concentric hypertrophy uniformly [9]. When present alongside other red flags, concentric biventricular hypertrophy with right ventricular (RV) wall hypertrophy can be suggestive of a storage etiology [15]. However, RV involvement in sarcomeric HCM is commonly observed and is therefore not pathognomonic [14]. The degree of hypertrophy may also provide an additive clue, though not independently discriminating. Extreme wall thickening ≥ 30 mm raises the overall clinical suspicion for a non-sarcomeric etiology when accompanied by electrophysiological abnormalities such as ventricular preexcitation [2]. However, extreme LVH of over 50 mm has also been observed in sarcomeric HCM [16]. Clearly, the morphological features of GSD cardiomyopathy and sarcomeric CM have significant overlap, which is why multimodal imaging and assessment of the GSD red flags are important to differentiate between GSD and sarcomeric cardiomyopathy [17].

Beyond morphology, speckle-tracking strain imaging is increasingly employed across GSD subtypes as both a diagnostic aid and a monitoring tool. Reduced global longitudinal strain (GLS) may precede overt systolic dysfunction not captured by conventional echocardiographic parameters, rendering assessment of GLS as a useful diagnostic tool in patients with preserved EF [17].

#### Advanced cardiac magnetic resonance imaging

Cardiac magnetic resonance imaging (CMR) provides critical tissue-level information that can differentiate GSD CM from sarcomeric HCM beyond what morphological imaging alone can achieve [18].

GSD patients have been found to have a significantly greater extent of late gadolinium enhancement (LGE) than those with sarcomeric HCM [18]. Nevertheless, GSD subtypes do not always produce LGE patterns distinct from sarcomeric HCM, with variability between patients diagnosed with the same GSD. Assessment of LGE pattern alone is therefore insufficient for subtype classification but can increase suspicion of a GSD etiology in many cases [18, 19].

Beyond LGE, T1 mapping and extracellular volume (ECV) fraction provide complementary tissue characterization. In sarcomeric HCM, native T1 is diffusely elevated, and ECV rises concordantly with progressive interstitial expansion and fibrosis [20]. In a select cohort, GSD patients had significantly higher global T1 and ECV when compared to sarcomeric HCM [18]. However, another case found a discordant pattern in which native T1 is elevated in LGE-negative myocardium while ECV remains relatively preserved [21]. Native T1 elevation without a corresponding rise of LGE or ECV suggests that intracellular pathology may alter myocardial relaxation properties before significant interstitial expansion occurs. Thus, discordance between native T1 values and LGE could be an indication of the early stage of disease progression [20].

In practice, CMR has become strongly recommended as a baseline assessment to differentiate between sarcomeric HCM and its mimics, including GSD cardiomyopathy. Thus, if ECG and echocardiogram results raise suspicion of a possible GSD, CMR is used for more advanced diagnostic imaging. CMR is equally valuable in monitoring disease progression. It can be used to measure the efficiency of enzyme replacement therapy (ERT) and gene therapies in treated patients as well [22].

CMR can provide disease-specific and subtype-sensitive myocardial tissue signatures in GSDs, but due to the variance of LGE, T1, and ECV findings between GSD patients and the lack of concrete GSD-specific CMR characterization, it is again most useful to integrate CMR within the full clinical picture of each patient.

#### Biochemical and metabolic screening

Biochemical testing offers a practical, low-cost screen that can serve as a preliminary diagnostic tool for suspected GSDs. First-tier tests include routine chemistry (basic metabolic panel (BMP), liver function tests (LFT), complete blood count (CBC)) and cardiac biomarkers (troponin, N-terminal pro-B-type natriuretic peptide (NT-proBNP), creatine phosphokinase) to assess organ involvement and cardiac severity, followed by disease-specific metabolic assays to help target a specific diagnosis [7, 23].

In contrast to GSD cardiomyopathies, routine serum creatine kinase (CK), liver enzymes, glucose, and lipid panels are uninformative as markers of primary sarcomeric disease [2]. Across GSD cardiomyopathies broadly, elevated serum CK reflects skeletal and cardiac muscle injury from glycogen accumulation and is a nonspecific but important screening marker; its presence alongside unexplained LVH substantially elevates suspicion for a metabolic etiology [9, 24]. Elevated LFT in a patient with cardiomyopathy can further direct the differential toward hepatic-involving GSDs, though to reliably localize organ involvement, additional clinical context is needed [5, 7, 9]. Fasting ketotic hypoglycemia and hypertriglyceridemia provide additional metabolic context that is largely absent in sarcomeric disease [1]. A key limitation of biochemical screening across the GSD spectrum is that not all subtypes produce consistently abnormal routine metabolites. Therefore, a normal metabolic screen does not exclude metabolic cardiomyopathy [4, 5, 7, 9]. Biochemical screening can provide important metabolic context that can be used to help reach a diagnosis.

### Definitive testing

#### Genetic testing, molecular diagnostics, and family screening

Given the high morbidity and the potential for sudden cardiac death (SCD) in metabolic cardiomyopathies, family screening is a fundamental component of management for GSDs. The Heart Failure Society of America (HFSA) Practice Guideline for Genetic Evaluation of Cardiomyopathy provides a structured framework for integrating genetic assessment into this process. Genetic testing is recommended for all patients with cardiomyopathy and should begin with the family member who has the most definitive diagnosis and/or the most severe disease manifestations [3]. The HFSA recommends obtaining a family history of at least three generations in all patients with a confirmed cardiomyopathy diagnosis, ideally on a team approach that includes genetic counselors [3]. All at-risk first-degree relatives are recommended to undergo baseline clinical phenotype screening for cardiomyopathy, and referrals to specialized centers should be completed for patients with genetic, familial, or unexplained cardiomyopathy. Following identification of a pathogenic or likely pathogenic variant, cascade genetic testing of at-risk relatives is strongly recommended [3]. This step is crucial because the first clinical manifestation of cardiomyopathy in a family member may be SCD [3]. Cascade screening serves a dual clinical function: identifying asymptomatic individuals with subclinical disease and detecting clinically unaffected carriers of pathogenic variants (genotype-positive, phenotype-negative individuals) [25]. Furthermore, early therapeutic intervention is critical to reduce mortality in certain GSD phenotypes [4]. From an economic standpoint, genetic testing and cascade screening are cost-effective in comparison to clinical screening [25]. The confirmatory genetic tests and disease-specific considerations are outlined in Table 1.

Cascade family screening following pathogenic variant identification is strongly recommended in all GSD subtypes with cardiac involvement. For X-linked conditions, all female first-degree relatives of affected males require evaluation [5]. For autosomal dominant conditions, all first-degree relatives should be offered testing [3].

**Table 1:** Cardiac and extracardiac manifestations, inheritance patterns, and genetic testing strategies across glycogen storage disease subtypes

Disease	Genetic etiology	Inheritance pattern	Cardiac manifestations	Extracardiac manifestations	Genetic testing strategies
Pompe Disease GSD IIa	*GAA* Acid alpha-glucosidase	Autosomal Recessive	**IOPD**: rapidly progressive massive LVH; LV outflow tract obstruction; cardiomegaly; Death in first two years without treatment, short PR interval, large QRS voltage **LOPD**: cardiac involvement less prominent; occasional conduction abnormalities; diastolic dysfunction	**IOPD**: “floppy baby” hypotonia, hepatomegaly, feeding difficulties, motor delays **LOPD**: proximal muscle weakness, respiratory insufficiency (third decade; sometimes earlier)	Acid alpha-glucosidase activity via biomarker testing; Genetic analysis of blood samples (*GAA*)
Danon Disease GSD IIb	*LAMP2* (*LAMP-2B* cardiac-predominant isoform)	X-linked Dominant	Massive LVH (commonly 35 ± 15 mm in males); Rapidly progressive systolic dysfunction; High burden of ventricular and SVA; WPW pattern on ECG; CMR: extensive patchy LGE, non-ischemic, predominantly subendocardial or transmural Females: later onset; subset develops severe rapidly progressive cardiomyopathy	Males: skeletal myopathy (↑ CK), intellectual disability Females: later, more variable; often cardiac-predominant Retinopathy	*LAMP2* sequencing; Genetic testing recommended even without family history (de novo mutations occur)
Cori/Forbes Disease GSD III	*AGL* Glycogen debranching enzyme (transferase + amylo-1,6-glucosidase activities)	Autosomal Recessive	LVH progressing to symptomatic HCM; dyspnea most common cardiac symptom Arrhythmia and symptomatic heart failure in a subset Glycogen in conduction system; ICD implanted in select cases	GSD IIIa (~ 85%): hepatic + skeletal muscle involvement GSD IIIb (~ 15%): hepatic involvement only Hypoglycemia (cornstarch supplementation required)	*AGL* gene sequencing
Andersen Disease GSD IV	*GBE1* Glycogen branching enzyme	Autosomal Recessive	Begins as HCM (cell swelling), progresses to DCM (cell death from mechanical strain) Congenital neuromuscular subtype: DCM in newborn period	**Fatal perinatal subtype**: fetal akinesia; neonatal death Congenital neuromuscular: hypotonia, respiratory distress; death in early infancy **Classic hepatic subtype**: hepatomegaly, cirrhosis, hypotonia; liver failure by age 5 without transplant **Non-progressive hepatic subtype**: hepatomegaly, liver dysfunction, myopathy Childhood neuromuscular subtype: rare, heterogeneous	*GBE1* gene sequencing
Tarui Disease GSD VII	*PFKM* Muscle isoform of phosphofructokinase	Autosomal Recessive	HCM from glycogen accumulation in cardiomyocytes (subset of patients) LV and IVS hypertrophy; abnormal cardiac conduction Documented progression: low-voltage ECG, supraventricular tachycardia, enlarged LA, mitral valve thickening, MR, diastolic dysfunction	Exercise intolerance, muscle cramps Myoglobinuria Compensatory hemolysis Later-onset de novo myasthenic syndrome	*PFKM* gene sequencing
Phosphorylase Kinase Deficiency GSD IX	*PHKA2*,* PHKB*,* PHKG2* Phosphorylase kinase subunits	X-linked for *PHKA-2* Autosomal Recessive for *PHKB* and *PHKG2*	Mild cardiomyopathy (↑ septal wall thickness) even in hepatic-presenting patients LVH in 2 of 18 patients in one natural history study Infantile cardiac subtype: marked myocardial glycogen accumulation, ventricular hypertrophy, cardiomegaly detectable in utero; large QRS, short PR, hypertrophic nonobstructive pattern on ECG	Classically a hepatic glycogenosis Hepatomegaly, liver dysfunction	*PHKA2*,* PHKB*,* PHKG2* sequencing
Glycogenin-1 deficiency GSD XV	*GYG1* Glycogenin-1 (self-glucosylating glycogen primer)	Autosomal Recessive	Ventricular dilatation, reduced EF, arrhythmogenic LV cardiomyopathy, ventricular tachyarrhythmias	Skeletal myopathy (variable; glycogen depletion in skeletal muscle) Significant inter-patient variability even with the same mutation	*GYG1* gene sequencing
PRKAG2 Syndrome	*PRKAG2* Regulatory γ2 subunit of AMPK	Autosomal Dominant	LVH; non-sarcomeric familial HCM Supraventricular tachyarrhythmias Ventricular preexcitation; WPW syndrome Chronotropic incompetence; high-degree AV block Premature pacemaker implantation frequently required	Rare skeletal myopathy found in 1463 A > T mutants	*PRKAG2* gene sequencing

Abbreviations: *GSD *Glycogen Storage Disease, *IOPD *Infant onset Pompe Disease, *LVH *left ventricular hypertrophy, *LV *left ventricle, *LOPD *Late onset Pompe Disease, *SVA *supra ventricular arrhythmias, *WPW *Wolff Parkinson White, *ECG *electrocardiogram, *CMR *cardiac magnetic resonance imaging, *LGE *late gadolinium enhancement, *CK *creatine kinase, *HCM *hypertrophic cardiomyopathy, *ICD *Implantable Cardioverter Defibrillator, *DCM *dilated cardiomyopathy, *IVS *intra ventricular septum, *LA *left atrium, *MR *mitral regurgitation, *EF *ejection fraction, *AV *atrio ventricular, *AMPK *adenosine monophosphate-activated protein kinase

#### Endomyocardial and extracardiac biopsy

The indications for tissue biopsy in metabolic cardiomyopathies have evolved substantially with the increasing availability and reliability of genetic testing, enzyme screening assays, and non-invasive imaging. Endomyocardial biopsy (EMB) is reserved for undiagnosed patients with cardiomyopathy whose noninvasive evaluation and molecular genetic testing remain inconclusive. It is important to note that EMB carries a higher procedural risk, and the decision to biopsy must be carefully weighed against the diagnostic yield, particularly given the increasing availability of non-invasive molecular diagnostics [26].

**Fig. 2:**
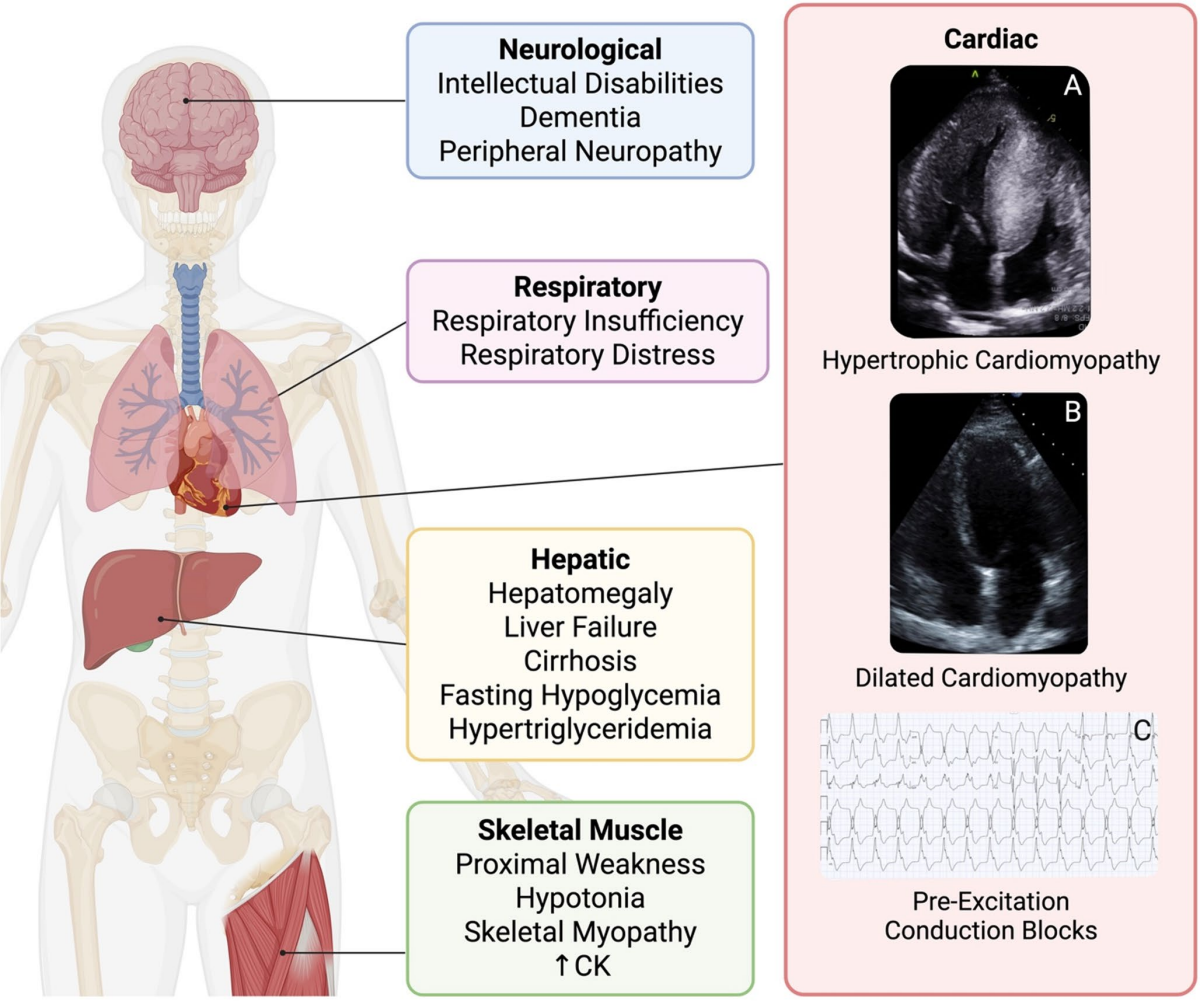
Multi-system manifestations of Glycogen Storage Diseases (GSDs) and representative phenotypes. The primary organ systems affected by GSDs are illustrated, encompassing neurological, respiratory, hepatic, and skeletal muscle involvement. Cardiac manifestations, which represent a leading cause of morbidity and mortality in many of these conditions, are highlighted with representative clinical findings. (**A**) Apical four-chamber echocardiogram from a 14-year-old male with Danon disease demonstrating marked hypertrophic cardiomyopathy (HCM), with severe concentric hypertrophy of the interventricular septum (IVS) and left ventricular (LV) free wall. (**B**) Echocardiogram from a 39-year-old female with Danon disease showing a DCM phenotype, characterized by increased LV end-diastolic diameter and reduced systolic function. (**C**) Twelve-lead electrocardiogram from a 20-year-old male with Danon disease demonstrating ventricular pre-excitation and conduction abnormalities. Figure created in BioRender. B, A. (2026) https://BioRender.com/n85hjyv. Abbreviation: CK = creatine kinase

Skeletal muscle, liver, and blood biopsy is available for select GSDs that present with extracardiac phenotypes [9]. However, biopsy interpretation is limited by sampling bias arising from spatial heterogeneity of glycogen accumulation, organ-to-organ variability, residual enzyme activity in later-onset phenotypes, and mosaic protein expression from random X-chromosome inactivation, contributing to possible false-negative results [5, 27]. These limitations can be mitigated by combining biopsy with enzymatic assays in multiple tissue sources and, definitively, by molecular genetic testing, which has largely supplanted invasive sampling as the diagnostic gold standard [9] (Fig. 2).

## Management of cardiomyopathy in glycogen storage diseases

### Conventional heart failure therapy

For heart failure with preserved ejection fraction (HFpEF), the evidence base has expanded substantially: following the EMPEROR-Preserved and DELIVER trials, sodium-glucose cotransporter 2 (SGLT2) inhibitors now carry Class I or IIa recommendations across major guidelines [28–31]. Diuretics remain a primary tool for symptom management [29]. However, these recommendations derive from pivotal trials that explicitly exclude patients with hypertrophic, infiltrative, and storage cardiomyopathies, including GSDs. HFpEF GDMT is therefore not validated in GSD populations and should not be applied uniformly without consideration of the individual disease phenotype of each patient [4, 29].

This caveat carries therapeutic consequences in GSDs that present with a hypertrophic, obstructive phenotype. In the setting of dynamic left ventricular outflow tract obstruction, guidelines caution that pure vasodilators and high-dose diuretics can worsen the outflow gradients and symptoms and may be relatively contraindicated. Non-vasodilating beta blockers, or non-dihydropyridine calcium channel blockers, are instead first-line [30]. A generic HFpEF strategy centered on aggressive diuresis may therefore be counterproductive in obstructive GSD cardiomyopathy, illustrating why phenotype-specific application is essential.

A further consideration across the GSD spectrum is the unpredictable phenotypic evolution from an HFpEF-dominant presentation to a heart failure with reduced ejection fraction (HFrEF) or mixed phenotype over time [4]. Once the left ventricular ejection fraction (LVEF) falls below 50%, GDMT for HFrEF, built on the four pillars of an angiotensin receptor-neprilysin inhibitor, an evidence-based beta blocker, a mineralocorticoid receptor antagonist, and an SGLT2 inhibitor, becomes appropriate [30]. This HFpEF to HFrEF transition also mandates reassessment of agents used during the hypertrophic phase: negative inotropes should be reconsidered, and cardiac myosin inhibitors discontinued, once systolic dysfunction develops [30]. Serial reassessment of cardiac function at defined intervals is therefore essential [4, 29, 30].

As in the case of HFpEF, HFrEF guidelines are not specific for GSDs and should therefore be applied with consideration of each GSD patient’s unique phenotype. Moreover, given the multi-system nature of most GSDs, tailored application of traditional therapies is crucial to account for the unique physiology of each GSD subtype, and more granular investigation within GSD populations is needed to define disease-specific treatment guidelines [4, 29].

### Arrhythmia and device therapy

Arrhythmia management in GSD cardiomyopathy is uniquely complex because multiple distinct mechanisms can coexist within the same patient. These mechanisms can include ventricular preexcitation, arrhythmias, chronotropic incompetence, and high-degree atrioventricular (AV) block [4, 13, 32]. The interaction between these mechanisms creates a high-risk arrhythmic substrate that would benefit from integrated electrophysiology (EP) assessment [4]. Importantly, established SCD risk stratification models developed for sarcomeric CM have not been validated in any GSD subtype. A low threshold for invasive EP evaluation and device therapy is therefore generally warranted when clinical red flags are present [4, 32].

The role of ambulatory (Holter) ECG monitoring is broader in GSD cardiomyopathy than in sarcomeric HCM. In HCM, Holter is principally used to detect nonsustained ventricular tachycardia (NSVT) for SCD risk stratification [30]. In GSD patients, the same recording must additionally survey for progressive conduction system disease, sinus node dysfunction, accessory-pathway-mediated tachycardia, and atrial fibrillation, each of which can independently dictate pacing, anticoagulation, ablation, or implantable cardioverter-defibrillator (ICD) therapy. Periodic monitoring with frequency adapted to symptom burden is therefore recommended across GSD subtypes [5, 13].

In the absence of GSD-specific risk scores, primary prophylactic ICD decisions extrapolate from HCM and DCM frameworks at a lower threshold, since arrhythmic event rates in Danon disease and PRKAG2 syndrome exceed those of unselected sarcomeric cohorts [5, 33]. For HCM-phenotype patients, conventional features (severe LVH, unexplained syncope, NSVT, family history of premature SCD, apical aneurysm) remain operative [30], with consensus support for an especially low threshold in Danon disease [5]. For PRKAG2 syndrome, formal ICD criteria have not been established, and individualized assessment is recommended [13]. For DCM-predominant phenotypes (female Danon patients, advanced GSD IV, GSD XV), conventional LVEF criteria apply, but contemporary guidelines support earlier ICD consideration when genetic or imaging features confer disproportionate risk [33].

For GSDs with a significant probability of malignant arrhythmic development, such as Danon disease and PRKAG2 syndrome, ICD placement is often pursued earlier than conventional HCM or DCM quantitative thresholds would suggest, with genotype and CMR fibrosis burden serving as primary determinants of timing [5, 13, 32, 33].

When ventricular preexcitation is present, catheter ablation may be attempted, but outcomes differ fundamentally from those in standard Wolff-Parkinson-White syndrome. Many GSD patients presenting with conduction abnormalities have experienced recurrence of abnormal conduction phenotype, due to the longitudinal progressive nature of these diseases [5, 13, 32].

### Mechanical circulatory support and advanced heart failure therapies

Given that many GSDs progress to advanced heart failure, ventricular assist devices and orthotopic heart transplant (OHT) are central to end-stage management of these diseases. The 2022 AHA/ACC/HFSA guidelines support the use of durable mechanical circulatory support (MCS) in advanced HFrEF with NYHA class IV symptoms despite GDMT, offering a reliable bridge to transplantation for GSD patients who present with a dilated cardiomyopathy or reduced EF phenotype [29]. However, most GSD patients with cardiac involvement express an HCM phenotype with preserved or mildly reduced LVEF [2, 4], which precludes durable MCS. To address this, the OPTN/UNOS Thoracic Organ Transplantation Committee issued the 2018 Review Board Guidance for Hypertrophic/Restrictive Cardiomyopathy Exemption Requests, which provides a non-device pathway to higher waitlist priority through National Heart Review Board approval. This allows GSD patients with HCM-predominant phenotypes to access Status 1–3 listing [34].

Standard transplant listing relies on cardiopulmonary exercise testing (CPET) thresholds derived from HFrEF cohorts (peak VO₂ ≤12 mL/kg/min on beta-blocker, or ≤ 14 mL/kg/min off beta-blocker) [29, 35]. The 2024 AHA/ACC HCM guideline holds that these cutoffs should not be the sole criterion for transplant eligibility in HCM-phenotype patients, who can decompensate rapidly despite preserved LVEF and peak VO₂ >14 mL/kg/min [30, 36]. Hence, the same principle applies when assessing GSD patients with HCM: red flags warranting earlier transplant evaluation include LVEF < 50% or new restrictive physiology, recurrent ventricular arrhythmia, new-onset atrial fibrillation, extensive CMR fibrosis, elevated natriuretic peptides, and declining CPET performance [36].

OHT has had favorable outcomes documented in Danon disease [37]. However, outcomes data for other GSDs are confined to isolated case reports. Because GSD patients are sometimes young at the time of first transplant, re-transplantation may eventually be required, underscoring the need for a curative treatment option like gene therapy.

### Gene therapy and molecular approaches

Gene replacement therapy has emerged as a promising disease-modifying approach for genetic cardiomyopathies, with adeno-associated viral (AAV) vectors becoming the dominant delivery platform. However, delivery vehicles face important constraints: AAVs have limited packing capacity, necessitating compact transgene cassettes and tissue-specific promoters to fit therapeutic cargo while preserving cardiac selectivity and minimizing off-target expression. The route of administration further complicates tissue targeting. Systemic intravenous delivery, required for diffuse myopathic diseases, demands high doses to overcome first-pass hepatic clearance and be effective for myopathic disease. However, the use of such high dosages provokes both an innate and adaptive immune response against the AAV capsid and its cargo, giving rise to a spectrum of immunotoxicities [38].

Together, these challenges highlight a need to address the concerns about targeted delivery and immunotoxicity. This has been and will be crucial in realizing the full potential of cardiac gene replacement therapy in the treatment of GSDs. Nevertheless, there has been much progress in this space, with many novel gene therapies emerging for GSDs in recent years (Table 2).

**Table 2:** Treatment strategies for cardiac glycogen storage diseases

GSD type	Standard therapy	Gene therapy/emerging trials
Pompe (GSD IIa)	**ERT**: Alglucosidase alfa (Myozyme/Lumizyme) is first-line [23]; Newly Food and Drug Administration-approved avalglucosidase alfa (Nexviazyme) and Cipaglucosidase alfa + miglustat (Pombiliti + Opfolda) is now used in practice as well [39] **Cardiac Management**: GDMT; standard cardiac drugs may be contraindicated in certain disease stages so management must be individualized [23]. **Advanced therapy**: Heart (or multi-organ) transplantation for refractory cardiomyopathy or heart failure.	**Heart-Directed Gene Therapy**: IOPD - GC301 (AAV9-coGAA, ChiCTR2200063229); single IV dose of 1.2 × 10¹⁴ vg/kg; cardiac improvement in 3/3 patients who survived to 1 year [40]. 1 patient died at week 21.
Danon (GSD IIb)	**Supportive cardiac care**: No disease-specific therapy exists. Use standard HF medications and manage arrhythmias with ablation or ICD implantation [5]. **Transplant**: Early heart transplantation is sometimes warranted due to rapid cardiomyopathy [5, 37].	**AAV9 gene therapy**: RP-A501 (AAV9-*LAMP2B*, Rocket Pharma) is in Phase 1/2 (NCT03882437). Interim data (up to 2-4.5 years follow-up) show cardiac *LAMP2* expression and stabilization/reversal of LVH in 6/7 treated patients [38, 41].
Cori/Forbes (GSD III)	**Diet**: High-protein diet (≈ 3 g/kg) with frequent feeds and uncooked cornstarch to prevent hypoglycemia [9]. **Supportive**: Standard HCM/HF management (beta-blockers, ACEi) as needed; no ERT available. Heart transplant in end-stage cases [9].	**Gene therapy**: No clinical trials to date.
Andersen (GSD IV)	**Cardiac Subtype**: Treat as DCM; standard HF meds, manage arrhythmias. In severe cardiomyopathy (often coexistent in classic GSD IV), heart transplant may be considered [42]. **Other**: Monitor for nutrition/coagulation issues; no medical ERT exists.	**Gene therapy**: No clinical trials to date.
*PRKAG2* Syndrome	HF and EP GDMT with advancement to MCS and OHT as needed [29].	**RNA silencing therapy**: AOC 1072 / ATR 1072 (Atrium Therapeutics) Antibody-oligonucleotide conjugate targeting transferrin receptor-1 to deliver siRNA selectively to cardiac muscle. ~85% knockdown of *PRKAG2* mRNA achieved in preclinical models without adverse ECG effects. Currently in IND-enabling studies; IND submission anticipated second half of 2026 [43, 44].

Abbreviation: *GSD *Glycogen Storage Disease, *ERT *enzyme replacement therapy, *FDA *Food and Drug Administration, *GDMT *guideline directed medical therapy, *IOPD *infant onset Pompe Disease, *HF *heart failure, *ICD *implantable cardioverter defibrillator, *LVH *left ventricular hypertrophy, *HCM *hypertrophic cardiomyopathy, *ACEi *angiotensin-converting enzyme inhibitors, *DCM *dilated cardiomyopathy, *EP *electrophysiology, *MCS *mechanical circulatory support, *OHT *orthotopic heart transplant, *siRNA *small interfering ribonucleic acid, *mRNA *messenger RNA, *ECG *electrocardiogram, *IND *investigational new drug

## Classification of metabolic cardiomyopathies

### Overview of glycogen storage diseases affecting the heart

The following sections review the cardinal features and manifestations of the principal GSD subtypes associated with cardiomyopathy.

### GSD II: Pompe disease

Pompe disease, also designated as GSD IIa, is an autosomal recessive lysosomal GSD caused by mutations in the *GAA* gene, resulting in acid alpha-glucosidase deficiency and consequent glycogen accumulation within lysosomes [45]. The penetrance for Pompe disease is not yet fully described [23]. The acid alpha-glucosidase enzyme is responsible for glycogen breakdown to glucose within the acidic lysosomal environment [23]. Two primary disease manifestations are recognized: infantile-onset Pompe disease (IOPD) and late-onset Pompe disease (LOPD), both involving *GAA* mutations but producing divergent phenotypes, with IOPD representing the more severe form [45]. The hypotonia, hepatomegaly, and feeding difficulties in infancy are extracardiac red flags for IOPD, while proximal muscle weakness and respiratory insufficiency in childhood or adolescence indicate LOPD. Additional indications of Pompe disease include a short PR interval with giant QRS voltages [46]. The global birth prevalence for IOPD and LOPD is 1.0 cases and 2.4 cases per 100,000 live births, respectively [46].

Dysfunctional acid alpha-glucosidase leads to vacuolization with periodic acid-Schiff (PAS)-positive lysosomes, which physically disrupts fibers within cardiac and skeletal muscle and leads to widespread oxidative and mitochondrial stress [23, 47, 48]. This is well supported by the fact that Pompe cardiomyocytes can exhibit widespread replacement fibrosis [49]. IOPD patients typically develop LVH within weeks of birth, accompanied by left ventricular outflow obstruction secondary to severe myocardial thickening and reduced lung volume from cardiomegaly. Diagnosis involves assessment of acid alpha-glucosidase activity through biomarker testing and genetic analysis of blood samples [45]. Prognosis and treatment response depend on the timing of diagnosis and treatment initiation; patients have trouble with speech, respiration and feeding due to muscle weakness. Routing surveillance to assess for new manifestations also serves as a key factor influencing disease progression [23]. Without intervention, IOPD death commonly occurs in the first two years of life [23, 45].

Alglucosidase alfa, the first approved enzyme replacement therapy (ERT), has dramatically improved survival and cardiac function in IOPD when initiated early [23]. Next-generation ERTs, avalglucosidase alfa (Nexviazyme), as well as combination therapy of cipaglucosidase alfa and miglustat (Pombiliti + Opfolda), have demonstrated clinically meaningful improvements in pivotal phase 3 trials (COMET and PROPEL, respectively). COMET met its non-inferiority endpoint while PROPEL did not achieve statistical superiority. Nevertheless, the promising results led to their approval for use [39]. As ERT reduces myocardial glycogen burden and LV mass regresses, the obstructive physiology driving contraindications of agents that reduce preload, afterload, or increase contractility may resolve. Cardiac management must therefore be regularly reassessed longitudinally to ensure that pharmacological therapies are properly tailored for each disease stage [23].

While recombinant human acid alpha-glucosidase (rhGAA) ERT revolutionized treatment for Pompe disease patients, rhGAA’s inability to cross the blood-brain barrier limits its use for treating neurological symptoms. Additionally, the formation of high sustained titers of anti-rhGAA after longitudinal use can reduce the efficiency of ERT therapy [40]. To overcome these limitations, gene therapy strategies are under active investigation. In the GC301 trial, four infants with IOPD received a single intravenous infusion of GC301, an AAV9 vector carrying codon-optimized human *GAA*. All three surviving patients demonstrated marked reductions in left ventricular mass index, with near normalization of myocardial hypertrophy in at least one patient, and EF was maintained throughout follow-up. Meaningful motor gains were observed in all survivors at one year. One patient died at week 21 following pneumonia-related respiratory failure after parents withdrew consent for invasive ventilation; no severe adverse events were attributed to GC301. These findings establish proof-of-concept for AAV9-mediated *GAA* replacement in IOPD, with particularly compelling cardiac outcomes following a single infusion [40].

### GSD IIb: Danon disease

GSD IIb, widely known as Danon disease, is an X-linked, multisystem autophagic storage disease caused by loss-of-function mutations in the *LAMP2* gene, encoding lysosomal-associated membrane protein type 2 (*LAMP-2*) [5]. Of the three *LAMP* isoforms (*LAMP-2 A*,* LAMP-2B*,* LAMP-2 C*), *LAMP-2B* is the cardiac-predominant isoform and mediates autophagosome-lysosome fusion [50]. This causes accumulation of cellular debris and glycogen within autophagic vacuoles, which physically disrupts muscle fibers and causes myofibrillar disarray. In addition to the mechanical myofibrillar impairments, Danon cardiomyocytes are rich with lipofuscin, suggesting extensive oxidative stress and mitochondrial damage [51, 52]. The result is severe LVH, rapidly progressive systolic dysfunction, and a high burden of ventricular and supraventricular arrhythmias [52]. Patients with Danon disease also classically develop skeletal myopathy and intellectual disabilities, which, along with HCM, constitute the classic triad [5]. Cardiomyopathy penetrance for males with the *LAMP2* mutation is extremely high, approaching 100% [53]. Males typically present with an earlier onset and more severe phenotype than heterozygous women [5, 54]. A study determined the average age for the onset of Danon disease symptoms was 12.1 ± 6.5 years in men and 28.1 ± 15 years in women, with cardiac symptoms noted earlier in men during their teens. Hypertrophic cardiomyopathy is predominant in men, while the dilated phenotype is more common in women; however, recent studies have noted the dilated disease among men and nearly equal rates of hypertrophic and dilated cardiomyopathy in women. The study also found that among patients with conduction abnormalities, the Wolff-Parkinson-White pattern on electrocardiograms was demonstrated mostly in men, with 68% compared to 27% of women. Cognitive disorders associated with Danon are also predominant among male patients compared to their female counterparts [52]. Danon disease accounts for about 4–6% of pediatric HCM cases [55].

The mean maximal LV wall thickness in male patients with LAMP2 mutations has been reported at 35 ± 15 mm in echocardiography [2, 24]. Speckle-tracking strain imaging reveals reduced GLS with apical sparing in Danon disease, and serial GLS measurement has been proposed as a monitoring tool for disease progression and therapeutic response [17]. CMR imaging in Danon disease shows extensive and patchy LGE in a non-ischemic, predominantly subendocardial or transmural distribution [56]. A distinctive T1 mapping/extracellular volume (ECV) discordance has been described, where native T1 is elevated while ECV fraction remains normal in areas without LGE, attributed to the intracellular nature of the autophagic vacuoles rather than extracellular expansion [57]. T2-weighted imaging may show hyperintense foci in areas of active myocardial injury, although hypointense foci are also observed [58].

Prognosis for Danon is poor without heart transplantation; however, frequent cardiac surveillance, ICD therapy, genetic counseling, comprehensive neuropsychological exams, and rehabilitation therapy are interventions that can help palliate symptoms [24]. Danon disease currently has no disease-specific therapies, with management primarily consisting of a multidisciplinary care approach for cardiomyopathy and Danon’s extracardiac manifestations [5]. The majority of male patients will reach end-stage heart failure, but only a minority will survive to a heart transplant [5, 37]. Those who undergo heart transplantation have been seen to have favorable outcomes [37]. One multi-center cohort study on OHT in 38 Danon disease patients found 5-year graft survival to be 87.1% [37]. Recent advances in gene therapy, however, offer a potential pathway toward durable genetic correction. AAV9-mediated LAMP2B delivery (RP-A501; Rocket Pharmaceuticals) has advanced into clinical trials. In a Phase 1 trial (NCT03882437), seven male patients aged 11–21 years received a single intravenous dose of RP-A501 with triple immunosuppression. Over 2-4.5 years of follow-up, cardiac *LAMP-2* expression was achieved, LV mass index decreased or stabilized, LVEF was preserved, and both troponin I and NT-proBNP declined. One patient with baseline LV dysfunction progressed to cardiac transplantation; all seven patients remained alive with resolved adverse effects. Adverse events included one grade 4 complement-mediated thrombotic microangiopathy and three grade 3 glucocorticoid-exacerbated myopathy events [38, 41].

The Phase 2 pivotal study (NCT06092034) is enrolling 12 male patients with co-primary biomarker endpoints of cardiac *LAMP-2* expression and LV mass reduction at 12 months, with secondary endpoints including troponin, NT-proBNP, and event-free survival [59]. In May 2025, the Food and Drug Administration (FDA) placed a clinical hold on the trial following a patient death attributed to capillary leak syndrome and acute systemic infection, with a novel C3 complement inhibitor in the pretreatment regimen identified as a suspected contributing factor. The hold was lifted in August 2025, with the FDA authorizing resumption at a recalibrated dose of 3.8 × 10¹³ GC/kg, administered sequentially with a minimum four-week interval between patients. As of that announcement, six patients had been treated in Phase 2 [60, 61].

### GSD III: Cori/forbes disease

Glycogen Storage Disease III, also known as Cori disease, Forbes disease, or glycogen debranching enzyme (GDE) deficiency, is an autosomal recessive disease characterized by pathogenic mutations in the *AGL* gene [62]. The penetrance for Cori/Forbes disease is not yet fully described, nor is there a clear genotype-phenotype correlation for all of the *AGL* variants [62]. If both parents are heterozygous, there is 25% chance of being affected, a 50% chance of being an asymptomatic carrier, and a 25% chance of inheriting neither of the familial variants [62]. Deficiency in GDE, which possesses transferase and amylo-1,6-glucosidase activities at two independent catalytic sites on a single polypeptide, leads to the accumulation of abnormally branched glycogen (limit dextrin) in the cytoplasm [9, 62]. This PAS-positive material is distributed within cardiomyocytes and extends to conduction cells throughout the heart. This accumulation leads to the development of moderate fibrosis in a subset of patients [63]. Two major subtypes exist: GSD IIIa, which accounts for approximately 85% of cases and involves both muscle and liver, and GSD IIIb, present in approximately 15% of cases and limited to hepatic involvement [63]. Overall, GSD III is relatively uncommon, with an estimated prevalence of 1 per 100,000 patients [62].

In GSD III, cardiomyocytes accumulate excess cytosolic glycogen, manifesting as PAS-positive, diastase-sensitive material distributed throughout the myocardium and extending to conduction cells of the sinoatrial node, bundle of His, and AV node. Notably, the development of moderate fibrosis in a subset of patients suggests that the pathophysiological mechanisms underlying this condition are diverse [63].

GSD III should be suspected in any individual with hepatomegaly in a child or adolescent, elevated CK, and transaminases with hypoglycemia. The primary cardiac manifestation of GSD IIIa is LVH; dyspnea has been reported as the most common cardiac symptom. Although less frequent, arrhythmia and symptomatic heart failure have been documented in a subset of GSD III patients [64]. In these instances, the ECG in GSD III typically shows LVH voltage criteria without delta waves or WPW pattern [9]. While GSD III patients generally have a normal life expectancy, muscular and cardiac symptoms, as well as liver fibrosis/cirrhosis and hepatocellular carcinoma, can negatively impact quality of life [62].

### GSD IV: Andersen disease

Andersen disease (GSD IV) is an autosomal recessive disease caused by pathogenic mutations in the *GBE1* gene, which encodes the glycogen branching enzyme [42]. Andersen disease penetrance differs as affected individuals are expected to manifest the same subtype of GSD IV, but age of onset and phenotype expression may differ [65]. Compound heterozygosity for pathogenic *GBE1* variants is common [65]. The glycogen branching enzyme catalyzes the transfer of alpha-1,4-linked glucosyl units to alpha-1,6 positions during glycogen synthesis; its deficiency results in the formation of abnormal, linear PAS-positive polyglucosan deposits that accumulate in the cytoplasm and cannot be effectively catabolized, leading to cell swelling and multi-organ dysfunction [65]. Additionally, there was a severe reduction in the density of myofibrils and mitochondria, with remaining myofibrils being pushed to the periphery of the cardiomyocytes [66]. GSD IV is rare, with a global incidence of about 1 per 600,000 to 800,000 people [65].

The primary cardiac phenotype is cardiomyopathy, which typically begins as hypertrophic, driven by cell swelling, and progresses to dilated due to cell death from sustained mechanical strain [42]. GSD IV encompasses several subtypes classified by age of onset and dominant organ involvement. The fatal perinatal neuromuscular subtype manifests with fetal akinesia deformation sequence, with death commonly occurring in the neonatal period. The congenital neuromuscular subtype presents with hypotonia, respiratory distress, and DCM in the newborn period, with death typically in early infancy. The classic progressive hepatic subtype may appear normal at birth but rapidly develops hepatomegaly, liver dysfunction, progressive cirrhosis, hypotonia, and cardiomyopathy, with liver failure and death by age five years without transplantation. The non-progressive hepatic subtype is characterized by hepatomegaly, liver dysfunction, and myopathy, but generally does not progress to end-stage liver disease. The childhood neuromuscular subtype is rare and clinically heterogeneous [65]. LGE may be paradoxically absent or heterogeneous despite significant cardiomyopathy, suggesting the dominant mechanism involves glycogen accumulation rather than replacement fibrosis detectable by gadolinium [67].

### GSD VII: Tarui disease

Glycogen Storage Disease Type VII (GSD VII), or Tarui disease, is a rare autosomal recessive disease caused by mutations in the *PFKM* gene, which encodes the muscle isoform of phosphofructokinase (PFK-M), a critical enzyme in glycolysis [68]. Due to its rarity, the penetrance of Tarui disease is not fully described.

While GSD VII primarily affects skeletal muscle metabolism, cardiac involvement has been documented, including LVH [69, 70]. A detailed longitudinal case study documented progressive cardiac abnormalities, including low-voltage ECG, supraventricular tachycardia, LVH, enlarged left atrium, mitral valve thickening, mitral regurgitation, and diastolic dysfunction, suggesting that GSD VII can pursue a multisystem course affecting both cardiac and neurological function in certain patients [69].

In GSD VII, evidence of electrophysiological abnormalities is currently limited to a single report, which tracked one patient over eight years. Findings included a low-voltage ECG pattern, left atrial enlargement, and episodes of supraventricular tachycardia [69]. While these observations might suggest a potential for glycogen-mediated atrial remodeling, they have not been corroborated by broader literature, making it difficult to classify them as characteristic features of the disease. GSD VII is very rare, limiting the availability of epidemiological studies to observe its prevalence. The prognosis for Tarui disease varies. In the infantile form, patients generally die within the first year of life. However, other patients with later onset can have a normal lifespan with exercise intolerance, muscle cramps, pain, and occasional nausea and vomiting on exertion [71].

### GSD IX: phosphorylase kinase deficiency

Glycogen Storage Disease Type IX (GSD IX) is a genetically heterogeneous disease caused by pathogenic variants in genes encoding subunits of phosphorylase kinase, most commonly *PHKA2*, *PHKB*, and *PHKG2* [72].

*PHKA2*-related liver PhK deficiency and *PHKA1*-related muscle PhK deficiency are inherited in an X-linked manner. Thus, hemizygous males who inherit a pathogenic variant are affected, while females are primarily heterozygous and very rarely homozygous [73]. The disease penetrance is therefore very high in males, but the phenotypic expression differs depending on the pathogenic variant; female disease penetrance varies based on X-linked inactivation [74].

*PHKB*-related liver and muscle PhK deficiency and *PHKG2*-related liver PhK deficiency are inherited in an autosomal recessive manner, and the disease penetrance for these variants has not been characterized [74]. Phosphorylase kinase is responsible for converting the inactive form of glycogen phosphorylase to the active phosphorylase form. Without this critical enzyme, PAS-positive inclusions of glycogen can accumulate in cardiomyocytes, hepatocytes, and skeletal myocytes [75, 76]. Though GSD IX is classically recognized as hepatic glycogenosis, cardiac involvement has been documented in rare case reports [72, 75]. Accordingly, information about the prevalence or incidence of muscle GSD IX is limited.

The cardiac phenotype of GSD IX is variable. In a large natural history study of GSD VI and IX, a GSD IXb patient was found to have mild cardiomyopathy, including increased septum wall thickness, on echocardiographic imaging, suggesting that myocardial involvement may occur even in patients initially identified through hepatic presentations [72]. A subsequent natural history study described left ventricular hypertrophy in 2 of 18 patients with GSD IX [75]. More severe cardiomyopathic phenotypes have been described in an isolated infantile case attributed to cardiac phosphorylase kinase deficiency. The report described GSDs confined predominantly to the heart, with marked myocardial glycogen accumulation, ventricular hypertrophy, and cardiomegaly detectable in utero; electrocardiographic findings included large QRS complexes, short PR interval, and a hypertrophic nonobstructive pattern [76]. The life expectancy for IX is normal, but outcomes are dependent on routine physical therapy, surveillance with imaging and echocardiogram, and feedings that are high in complex carbohydrates and protein to combat hypoglycemia [73].

### GSD XV: glycogenin-1 deficiency

Glycogen Storage Disease Type XV (GSD XV) is an autosomal recessive disease caused by pathogenic variants in *GYG1*, encoding glycogenin-1, a self-glucosylating protein that forms the primer required for glycogen chain elongation. The disease penetrance has not been fully explored for GSD Type XV, possibly due to the rarity of the disease [77]. When glycogenin-1 is dysfunctional, early glycogen biosynthesis is impaired, leading to cardiomyopathy with abnormal accumulation of glycogen within the nuclei of cardiomyocytes and glycogen depletion in skeletal muscle [77, 78].

GSD XV is commonly associated with an arrhythmogenic LV cardiomyopathy phenotype characterized by extensive myocardial fibrosis, life-threatening ventricular tachyarrhythmias, and thromboembolic events, constituting a highly arrhythmogenic substrate [10]. However, clinical cardiac phenotypes of GSD XV vary significantly, even between patients with the same mutation. In one series, three unrelated adult men with biallelic *GYG1* variants developed cardiac disease manifesting as ventricular dilatation, reduced EF, and extensive LGE on CMR; two of these patients required cardiac transplantation [78]. GSD XV is ultra-rare; studies to measure its prevalence or incidence have not yet been carried out. The prognosis for GSD XV varies, with some experiencing progressive liver cirrhosis leading to death in childhood unless liver transplantation is performed, while others with the adult-onset variant show slowly progressive myopathy with muscle weakness [78]. There are also multiple cases of patients with *GYG1*-associated cardiomyopathy that eventually progress to heart failure [78].

### *PRKAG2* syndrome

*PRKAG2* syndrome is an autosomal dominant disease caused by gain-of-function pathogenic mutations in the *PRKAG2* gene, which encodes the regulatory gamma-2 subunit of AMP-activated protein kinase (AMPK). The disease penetrance is 100% if the individual inherits the variant gene [79]. The gamma-2 subunit normally senses cellular energy status through the detection of ATP, ADP, and AMP levels and coordinates glucose uptake accordingly. Pathogenic mutations lead to AMPK dysfunction, altering the myocyte glucose uptake and metabolism, causing the deposition of glycogen and amylopectin, which accumulate to form massive PAS-positive glycogen-filled cytoplasmic vacuoles, leading to mechanical displacement of cardiac muscle fibers without pronounced lysosomal rupture or myofiber disarray [18, 80, 81]. The cardiac phenotype observed in *PRKAG2* syndrome is primarily driven by glycogen accumulation, but the presence of extensive LGE suggests that fibrosis may also contribute [81]. *PRKAG2* Syndrome is very rare, and its prevalence has not yet been determined, but one study found that about 1 in 100 cases of cardiomyopathy were caused by a *PRKAG2* mutation [13].

AMPK is highly expressed in cardiac tissue; therefore, this mutation drives a spectrum of cardiac disease, including supraventricular tachyarrhythmias, chronotropic incompetence, and non-sarcomeric familial HCM associated with WPW syndrome [13, 81]. In the multicenter Lopez-Sainz cohort (*n* = 90), the following events were documented over a median follow-up of approximately 6 years: 71% of individuals had LVH, 21% of patients had de novo pacemaker implantation, 29% had new-onset atrial fibrillation, and 8% had SCD [80]. Regular ECG and Holter monitoring, with frequency adapted to individual symptoms burden, is recommended [13]. *PRKAG2* syndrome may be distinguished from Danon disease by its usual absence of extracardiac features in the majority of cases [13]. Rare skeletal myopathy has been observed in 1463 A > T mutants [13]. The prognosis for *PRKAG2* Syndrome is poor with a high rate of complications, including juvenile onset of conduction disease, advanced HF, and in some cases, lethal arrhythmias [80].

An antibody-oligonucleotide conjugate (AOC 1072/ATR 1072; Avidity Biosciences, now Atrium Therapeutics) targeting the transferrin receptor-1 has been developed to deliver siRNA selectively to cardiac muscle, achieving approximately 85% knockdown of PRKAG2 mRNA in non-human primates and reduced glycogen accumulation in a murine *PRKAG2* variant model without adverse electrocardiographic effects [43]. These preclinical findings support an anticipated first-in-human IND submission in the second half of 2026 [44].

## Conclusion

Cardiomyopathies arising from GSDs are a frequently underrecognized spectrum of inherited heart disease. Early and accurate diagnosis is imperative, as delayed recognition condemns patients to preventable morbidity and premature death. This set of progressive cardiomyopathies may masquerade as sarcomeric HCM, so integration of clinical red flags, advanced cardiac imaging, genetic testing, and tissue biomarkers is essential for accurate diagnosis.

Despite meaningful advances, critical knowledge gaps remain. Disease-specific heart failure guidelines distinct from those developed for sarcomeric HCM are lacking, and the natural history of many rare GSD subtypes is incompletely characterized. Registry-based longitudinal datasets and multicenter collaborative frameworks can address deficiencies and enable adequately powered therapeutic trials. Personalized medicine approaches informed by genotype, sex, and modifier genetic factors will be essential as well.

The integration of genetics into cardiomyopathy and heart failure decision-making is now fundamental to precision cardiovascular care. As gene therapy programs mature across GSD subtypes, the prospect of durable molecular correction is increasingly within reach, offering hope for a patient population historically with few therapeutic alternatives.

## Data Availability

No datasets were generated or analysed during the current study.
